# Skin Diseases and Syphilis

**Published:** 1895-08

**Authors:** 


### *SELECTIONS AND ABSTRACTS.*

### SKIN DISEASES AND SYPHILIS.

Edited by Dr. M. B. Hutchins.

### The Permanent Destruction of Superfluous Hair.

The presence of few or many coarse hairs upon a lady's face, either on the lip, chin, cheeks, or in a mole, is often a source of humiliation and unhappiness to the victim. My observation is, that their occurrence is more frequent in the brunette type than in the blonde, though the natural, fine lanugo hairs of either type may become unpleasantly conspicuous.

Many sufferers seek to remove the deformity by pulling out the hairs by the root, a procedure which is very painful, and only serves to render them coarser. Others use various advertised depilatories, while some are so seriously afflicted as to be driven to regular shaving. Various hair solvents have been used, but their action is uncertain, and they may not only produce an inflammation of the skin, but also stimulate the hair to more vigorous growth.

The only method of permanently destroying these hairs, without injury or disfigurement, is that of electrolysis. About twenty years ago Dr. Michel, an oculist of St. Louis, introduced this method for trichiasis; Dr. Hardaway, of the same city, afterwards adopting it in dermatology.

The operation has been varied in its minor details by different dermatologists and practitioners, and no doubt it has also so often been unskillfully done as to bring it into disrepute.

My own method may briefly be detailed here. The current from five to seven galvanic cells is used, according to the condition of the battery and the requirements of the case. To the positive pole the ordinary sponge electrode is attached, while the needle holder, containing a fine steel needle, is attached to the negative pole. If the needle is attached to the positive pole the pain of the operation seems intensified, and a blackish point is produced, which persists for some time. The needle holder is about the size of a pencil, about five inches long, and has a spring upon it for the closing of the circuit. Most operators let the patient control this by pressing the sponge electrode in the hand, but having the control of the current entirely in the operator's hands—as the spring permits—has obvious advantages. The patient is placed upon the operating lounge before a good light, the operator sits to the right, the sponge electrode is thoroughly wet with water and firmly grasped in the patient's palm. The needle is then gently passed along the hair, in the follicle, until slight resistance, the bottom of the follicle, is felt. This is the site of the hair papilla, which must be destroyed. The spring is then pressed, upon which slight frothing occurs around the needle, and the surrounding skin is blanched. If the exact course of the hair is followed the needle enters more easily, the pain of the current seems less, and the frothing more. After five to thirty seconds, depending upon the size of the hair, the circuit is broken by releasing the spring, and the needle withdrawn.

If the hair attachments have been destroyed, the hair will come out with no resistance to the epilating forceps. If it resists, the current must be reapplied. Hairs in moles may be destroyed in the same way, after which the mole will gradually atrophy, electrolysis having this effect when passed through them. The first sensation from the current suggests a bee sting, rather startling to the patient, and then the needle feels hot in the skin. But the patient will "suffer to be beautiful," and, during my four or five years of this work I have never had a patient decline to go on with the treatment.

Fifty to one hundred hairs, according to their size, may be removed in an hour. An hour is long enough to strain the patient's nerves and the operator's eyes, though I have worked nearly three hours at one sitting, and once treated a patient an hour each morning and afternoon for five days, removing in that time over seven hundred hairs.

Only those hairs which are easily seen should be treated, the position of the root of the very small ones being too uncertain. I

have had no ill effects from removing the hairs side by side, though there is danger of setting up an inflammation, or producing superficial scarring. The skin appears red for a few hours after the treatment, and a fine scale often marks the site from which each hair has been removed, remaining a few days.

The success of the treatment depends upon the skill of the operator; the percentage of regrowth is from five up. It is well to keep account of the hairs removed, for record and future comparison. In estimating regrowths, it should be remembered that the lanugo hairs may, in time, become coarse. The root of some hairs may be so bent or curled as to render difficult the proper location of the papilla.

While the method described is tedious and painful, it is the only certain means of getting free of the hairs, and it must continue to be used until something better is found. No mode of anesthesia is practicable, owing to the time of the operation and the extent of the involved areas.

Any physician with the proper apparatus, "a steady hand, good eyesight, patience," and the time, can easily learn to do the work, but he will find that considerable practice is necessary to becoming adept in it.—Dr. M. B. Hutchins, in *Alabama Medical and Surgical Age*, July 1, 1895.

---

### A Case of Urticaria Pigmentosa.

Dr. L. Derville. (*Journal des Sciences Médicales de Lille*, February, 1894.) The patient was a girl aged ten months. The affection first appeared at the age of six weeks, as an eruption of itching papules, most abundant on the back. By the appearance of successive crops the eruption became rapidly almost general. When first seen, at the age of ten months, the back was thickly covered with slightly raised oval patches of a brownish color, with here and there maculæ. On the front of the chest the eruption was less abundant, and maculæ predominated. The hands and feet and the mucous membrane of the mouth were free. On the scalp, face, and neck were pale *café-au-lait* colored non-raised patches.

Slightly enlarged glands in neck, axillæ and groins.

On local irritation, the papules became tumid, and resembled urticarial wheals. When the child cried, or on removing the clothes, the whole skin surface became congested, with severe itching.

The urticarial symptoms were relieved by treatment, but the eruption itself remained unchanged.—*British Journal of Dermatology*, June, 1895.

